# Design and Performance of a New Severity Score for Intermediate Care

**DOI:** 10.1371/journal.pone.0130989

**Published:** 2015-06-29

**Authors:** Félix Alegre, Manuel Fortún Landecho, Ana Huerta, Nerea Fernández-Ros, Diego Martínez-Urbistondo, Nicolás García, Jorge Quiroga, Juan Felipe Lucena

**Affiliations:** 1 Division of Intermediate Care and Hospitalists Unit, Department of Internal Medicine, Clínica Universidad de Navarra, Pamplona, Navarra, Spain; 2 Instituto de Investigación Sanitaria de Navarra (IdiSNA), Pamplona, Navarra, Spain; 3 Centro de Investigación Biomédica en Red de Enfermedades Hepáticas y Digestivas (CIBEREHD), Madrid, Spain; University of Florida College of Medicine, UNITED STATES

## Abstract

**Background:**

Application of illness-severity scores in Intermediate Care Units (ImCU) shows conflicting results. The aim of the study is to design a severity-of-illness score for patients admitted to an ImCU.

**Methods:**

We performed a retrospective observational study in a single academic medical centre in Pamplona, Spain. Demographics, past medical history, reasons for admission, physiological parameters at admission and during the first 24 hours of ImCU stay, laboratory variables and survival to hospital discharge were recorded. Logistic regression analysis was performed to identify variables for mortality prediction.

**Results:**

A total of 743 patients were included. The final multivariable model (derivation cohort = 554 patients) contained only 9 variables obtained at admission to the ImCU: previous length of stay 7 days (6 points), health-care related infection (11), metastatic cancer (9), immunosuppressive therapy (6), Glasgow comma scale 12 (10), need of non-invasive ventilation (14), platelets 50000/mcL (9), urea 0.6 g/L (10) and bilirubin 4 mg/dL (9). The ImCU severity score (ImCUSS) is generated by summing the individual point values, and the formula for determining the expected in-hospital mortality risk is: e^ImCUSS points*0.099 – 4,111^ / (1 + e^ImCUSS points*0.099 – 4,11^1). The model showed adequate calibration and discrimination. Performance of ImCUSS (validation cohort = 189 patients) was comparable to that of SAPS II and 3. Hosmer-Lemeshow goodness-of-fit C test was χ^2^ 8.078 (p=0.326) and the area under receiver operating curve 0.802.

**Conclusions:**

ImCUSS, specially designed for intermediate care, is based on easy to obtain variables at admission to ImCU. Additionally, it shows a notable performance in terms of calibration and mortality discrimination.

## Introduction

Worldwide health care institutions try to give care based on best-practice models with cost-effectiveness. However, hospitalized patients are becoming complex and many of them exceed monitoring and nursing care available in conventional hospitalization wards. In this scenario, intermediate care units (ImCU) may provide a rational and proportional treatment between the intensive care units and the general ward [1–11].

The characteristics of ImCUs depend on resource availability, institutional infrastructure and the parent health care system. They can function as step-up or step-down units, or provide specialty care for cardiac, neurologic, respiratory or surgical conditions. Multipurpose ImCUs need characterization of the admitted patients in order to assess their illness severity and prognosis. Multiple severity scores have been designed and widely described in patients admitted to intensive care units (ICU) [12–21], but information in the setting of ImCU is scarce [1,5,8–9,11]. Moreover, the performance of the Simplified Acute Physiology Score II (SAPS II) [13] and the Simplified Acute Physiology Score 3 (SAPS 3) [14] showed conflicting results in intermediate care [5]. In addition, the collection of data for the calculation of these illness severity scores is time consuming.

The purpose of the present study is to design, based on simple variables, a new severity-of-illness scoring system for intermediate care, and to assess its performance in a single centre ImCU.

## Patients and Methods

In order to develop the ImCU Severity Score (ImCUSS) we performed a retrospective observational study, with data collected from April 2006 to December 2013 in a single academic medical centre in Pamplona, Spain. The ImCU is a 9-bed unit adjacent to, but independent from, the mixed ICU. Each bed is equipped with a continuous telemetry, pulse oximetry, non-invasive arterial blood pressure, central venous pressure monitoring, and non-invasive pressure support ventilation. The signals are relayed to a central monitoring station and the nurse:patient ratio is 1:3. The ImCU infrastructure (beds, technical resources and nursing staff) is shared with the Stroke Unit and the Coronary Care Unit. The ImCU rounding team involves a nurse, the hospital pharmacist, the ImCU resident, the specialist or surgeon and the attending hospitalist. The hospitalist is responsible for admission and discharge of all ImCU patients. Admission and discharge criteria were set according to previous guidelines defined by The American College of Critical Care Medicine [22]. Exclusion criteria included age less than 18 years old, severe respiratory failure at imminent risk of requiring intubation, status epilepticus, and catastrophic brain illness.

From April 2006 to December 2013, every consecutive patient admitted to the ImCU was evaluated. ImCU readmissions and low risk patients admitted exclusively for drug administration and desensitization were excluded from data analysis. Demographics, past medical history, reasons for admission, physiological parameters at the time of admission and during the first 24 hours of ImCU stay, laboratory variables, location at discharge and survival to hospital discharge were recorded. SAPS II and SAPS 3 were also calculated according to standard coefficients.

Statistical analysis was performed using SPSS for Windows, version 20.0 (SPSS Inc, Chicago; IL). Continuous variables were reported as mean ± standard deviation or median (25%-75% interquartile range).

Logistic regression was used to develop an in-hospital mortality prediction model for patients admitted to our ImCU. Variables to be included in the multivariable model were determined by using univariable logistic regression (p value in the univariable model ≤0.20). The maximum likelihood method was applied to estimate model coefficients, and these were then used to develop our new severity score. We assigned integer point values to each predictor variable from the multivariable logistic regression model (the coefficients of these variables were transformed by multiplying them by 10 and rounding to the nearest integer), thus generating the ImCUSS. The expected mortality rates were calculated using the ImCUSS as the only variable in another logistic regression model.

The ImCUSS was derived using a random sample of 75% subjects from the cohort, and then validated in the remaining 25%. For both instances, the calibration and discrimination performances were evaluated with the Hosmer-Lemeshow goodness-of-fit (GOF) C test and the area under the receiver operator curve (AUROC), respectively.

### Ethics statement

The study protocol was approved by the Institutional Review Board (IRB) at the Clínica Universidad de Navarra (ref. 129/2010). The IRB waived the need for informed consent, because it is an observational non-interventional study, and also because it did not interfere with decisions related to patient’s care. The study has been performed in accordance with the ethical standards laid down in the 1964 Declaration of Helsinki and its later amendments. Patient information was anonymized and de-identified prior to analysis.

## Results

During the study period, 1112 patients were admitted to the ImCU. Of these, 369 were excluded: 70 low-risk patients (drug administration and desensitization), 200 readmissions, and 99 patients for missing variables. Therefore, 743 patients were included in data analysis.

Patient characteristics, reasons for admission, and relevant data regarding main differences between survivors and deaths are summarized in Table 1. The mean age was 67 years with 62% male. The patients were admitted from the general ward (50.4%), the emergency room (27.1%), the ICU (13.1%), the operating room (6.0%) and from other hospital wards (3.4%). Reasons for admission were primarily medical (88.7%), with respiratory failure (34.2%) and sepsis (21.9%) as the leading causes. The median length of ImCU stay was 4 (2–7) days.

**Table 1:** Patient characteristics and complementary data.

		Overall population (n = 743)	Survivors (n = 597)	Deaths (n = 146)	p *
Sex (male) (%)		62.2	62.5	61.0	0.734
Age (years)		67 ± 15	67 ± 15	67 ± 13	0.782
Location prior to admission (%)					**<0.001**
	*Emergency room*	*27*.*1*	*29*.*3*	*17*.*8*	
	*General ward*	*50*.*4*	*47*.*1*	*64*.*4*	
	*ICU* ^*1*^	*13*.*1*	*12*.*7*	*14*.*4*	
	*Operating room*	*6*.*0*	*7*.*5*	*0*.*0*	
	*Other centre*	*3*.*4*	*3*.*4*	*3*.*4*	
Type of admission (%)					**<0.001**
	*Medical*, *non-urgent*	*10*.*6*	*9*.*2*	*16*.*4*	
	*Medical*, *urgent*	*78*.*1*	*77*.*1*	*82*.*2*	
	*Surgical*	*11*.*3*	*13*.*7*	*1*.*4*	
Reason for admission (%)					**<0.001**
	*Sepsis*	*21*.*9*	*20*.*1*	*29*.*5*	
	*Respiratory failure*	*34*.*2*	*31*.*7*	*44*.*5*	
	*Cardiovascular*	*11*.*6*	*12*.*7*	*6*.*8*	
	*Gastrointestinal*	*9*.*8*	*10*.*4*	*7*.*5*	
	*Neurologic*	*4*.*8*	*4*.*9*	*4*.*8*	
	*Monitoring*	*14*.*0*	*16*.*2*	*4*.*8*	
	*Other*	*3*.*7*	*4*.*0*	*2*.*1*	
Do-not resuscitate orders (%)		19.7	13.4	45.2	**<0.001**
ECOG (%)					**<0.001**
	*0 and 1*	*28*.*7*	*32*.*8*	*11*.*6*	
	*2*	*42*.*9*	*44*.*1*	*38*.*4*	
	*3 and 4*	*28*.*4*	*23*.*1*	*50*.*0*	
Infection at admission (%)					**<0.001**
	*Acquired in the community*	*18*.*2*	*20*.*8*	*7*.*5*	
	*Health-care related*	*49*.*5*	*42*.*2*	*79*.*5*	
Previous length of stay (days)		3 (0–8)	2 (0–7)	6 (1–12)	0.059
Metastatic cancer (%)		25.6	19.6	49.5	**<0.001**
Immunosuppressive therapy (%)		44.4	38.5	68.5	**<0.001**
Cirrhosis (%)		9.2	7.0	17.8	**<0.001**
COPD ^2^ (%)		19.5	21.1	13.0	**0.027**
Hypertension (%)		51.5	53.1	45.2	0.087
Systolic BP ^3^ (mmHg)		117 ± 27	118 ± 27	115 ± 25	0.209
Heart rate (lpm)		93 ± 21	91 ± 20	101 ± 21	**<0.001**
Supplemental oxygen support (%)					**<0.001**
	*None*	*36*.*0*	*40*.*2*	*19*.*2*	
	*Oxygen support without NIV* ^*4*^	*38*.*4*	*38*.*7*	*37*.*0*	
	*NIV ^4^*	*25*.*6*	*21*.*1*	*43*.*8*	
Glasgow comma scale ≤12 (%)		8.5	7.4	13.0	**0.028**
pCO2 (mmHg)		41 ± 12	42 ± 12	38 ± 12	**<0.001**
Lactate ≥3 mmol/L (%)		11.7	9.2	21.9	**<0.001**
Haemoglobin (g/dL)		10.8 ± 2.3	10.9 ± 2.4	10.3 ± 1.9	**0.005**
Leukocytes (/mcL)		10.7 ± 7.0	10.5 ± 6.6	11.7 ± 8.2	0.103
Platelets (/mcL)		222 ± 148	229 ± 143	194 ± 164	**0.018**
Pro-thrombin time (%)		79.1 ± 42.0	81.0 ± 45.1	71.5 ± 24.8	**0.017**
C-reactive protein (mg/dL)		12.4 ± 11.3	12.0 ± 11.3	14.1 ± 11.5	0.056
Creatinine (mg/dL)		1.4 ± 1.1	1.4 ± 1.1	1.6 ± 1.2	0.182
Urea (g/L)		0.60 ± 0.42	0.57 ± 0.39	0.75 ± 0.47	**<0.001**
RIFLE score (%)					**0.026**
	*Risk*	*10*.*6*	*9*.*9*	*13*.*7*	
	*Injury*	*5*.*8*	*5*.*4*	*7*.*5*	
	*Failure*	*4*.*3*	*3*.*4*	*8*.*2*	
	*Loss*	*0*.*3*	*0*.*2*	*0*.*7*	
	*ESRD* ^*5*^	*2*.*2*	*2*.*0*	*2*.*7*	
Albumin (g/dL)		2.8 ± 0.8	2.8 ± 0.8	2.5 ± 0.7	**<0.001**
Bilirubin (mg/dL)		2.2 ± 5.0	1.8 ± 4.1	3.9 ± 6.9	**0.001**
Lowest SPB ^6^, 24 hours (mmHg)		99 ± 19	100 ± 19	93 ± 19	**<0.001**
Vasoactive drugs, 24 hours (%)		17.6	16.2	23.3	**0.045**
Blood supplements, 24 hours (%)		34.7	31.5	47.9	**<0.001**
Urine output, 24 hours (mL)		1771 (1135–2690)	1850 (1179–2700)	1585 (894–2577)	**0.017**
SAPS II score (expected mortality rate)		33.1 ± 12.9 (19.2%)	30.6 ± 11.6 (15.5%)	43.6 ± 12.6 (34.3%)	**<0.001**
SAPS 3 score (expected mortality rate)		60.3 ± 14.0 (38.2%)	57.5 ± 13.3 (33.6%)	71.7 ± 10.8 (57.5%)	**<0.001**
Location at discharge (%)					**<0.001**
	*General ward*	*77*.*4*	*85*.*8*	*43*.*6*	
	*ICU * ^*1*^	*10*.*0*	*6*.*0*	*26*.*3*	
	*Operating room*	*3*.*7*	*3*.*9*	*2*.*7*	
	*Other centre*	*1*.*8*	*2*.*3*	*0*.*0*	
	*Home*	*1*.*7*	*2*.*0*	*0*.*0*	
	*Death (in ImCU* ^*7*^ *)*	*5*.*4*	*0*.*0*	*27*.*4*	

* All the remaining variables including diabetes, dyslipidemia, heart failure, previous coronary heart disease, heart arrhythmia, neurologic illness, body mass index, temperature, pH, sodium, potassium, magnesium and GPT were not statistically significant. Some variables described as continuous were also studied as polychotomous and dichotomous ones.

^1^ ICU: Intensive Care Unit.

^2^ COPD: Chronic Obstructive Pulmonary Disease.

^3^ BP: Blood Pressure.

^4^ NIV: Non-invasive Ventilation.

^5^ ESRD: End-Stage Renal Disease.

^6^ SBP: Systolic Blood Pressure.

^7^ ImCU: Intermediate Care Unit.

As summarized in Table 1, factors significantly related to in-hospital mortality were SAPS II and 3 scores, the presence of metastatic cancer (includes solid organ metastatic cancer and haematological cancer), immunosuppressive therapy, do-not-resuscitate (DNR) orders, ECOG status, health-care related infection, need of non-invasive mechanical ventilation, impaired neurologic status assessed by Glasgow comma scale, cirrhosis, acute renal failure measured by RIFLE score, tachycardia, decreased urine output in the first 24 hours of ImCU admission, and need of vasoactive drugs and blood transfusions in the same period. In addition, some laboratory variables like decreased pCO2, haemoglobin, platelets and albumin, prolonged pro-thrombin time and increased lactate, C-reactive protein, urea and bilirubin were also related with in-hospital mortality.

### Design and performance of the ImCUSS

The final multivariable model obtained from the derivation cohort (554 patients) contained only 9 variables that were obtained at admission to the ImCU: previous length of stay ≥7 days, health-care related infection, metastatic cancer, immunosuppressive therapy (includes steroids and other immunosuppressive drugs), Glasgow comma scale ≤12, need of non-invasive mechanical ventilation, platelets ≤50000/mcL, urea ≥0.6 g/L and bilirubin ≥4 mg/dL. No continuous variables were included, as some of them were transformed into dichotomous variables to simplify the model (conversion of polychotomus and continuous variables from the univariable analysis to dichotomous ones in the final multivariable analysis was made based on sensitivity analysis regarding different groups obtained for each variable, and favouring previous cut-offs described in the literature when possible). Parameters that were measured in the 24 hour interval after admission to the ImCU were also excluded, in order to make the score able to be calculated at admission to the ImCU and to avoid the potential Boyd and Grounds effect [23]. Additionally, we did not include variables that could have in our data a subjective component, such as ECOG scale (it was collected retrospectively in our series). Both DNR orders and the presence of solid organ metastatic and/or haematological cancer were significantly related to in-hospital mortality in the univariate analysis. Nonetheless, in order to avoid collinearity (65.4% of patients with DNR orders had also metastatic cancer), we excluded DNR for the final multivariate model.

The logistic regression model coefficients, SE, odds ratios, the 95% CI for the odds ratios, p values, and the point value for each of the predictor variables are shown in Table 2. Model performance was adequate, with Hosmer-Lemeshow GOF C test showing χ^2^ 13.039, degrees of freedom 8, p = 0.111. p-value is greater than 0.05, suggesting that the model is well calibrated and predicts probabilities that reflect the true mortality experience of the data. The bootstrapping simulation validates that the developed model has good calibration as 974 of the 1000 Hosmer-Lemeshow GOF p values (97.4%) were greater than 0.05.

**Table 2:** Mortality prediction model—using maximum likelihood estimation logistic regression.

Variables (at admission)	Coefficient	SE *	OR ₸	95% CI ³	p	Point values
Previous length of stay ≥7 days	0.601	0.261	1.82	1.09–3.04	0.021	6
Health-care related infection	1.093	0.289	2.98	1.69–5.26	<0.001	11
Metastatic cancer	0.869	0.293	2.38	1.34–4.23	0.003	9
Immunosuppression	0.609	0.288	1.84	1.05–3.24	0.035	6
Glasgow comma scale ≤12	1,030	0.398	2.80	1.28–6.11	0.010	10
NIV ^1^	1.353	0.266	3.87	2.30–6.52	<0.001	14
Platelets ≤50000/mcL	0.878	0.412	2.41	1.07–5.39	0.033	9
Urea ≥0.6 g/L	1.020	0.263	2.77	1.66–4.64	<0.001	10
Bilirubin ≥4 mg/dL	0.940	0.370	2.56	1.24–5.29	0.011	9
Constant	-4.122	0.358			<0.001	

* SE: Standard Error

^₸^ OR: Odds Ratio

^3^ CI: Confidence Interval

^1^ NIV: Non-invasive ventilation

The ImCUSS is generated by summing the individual point values (Table 2) based on the patients’ status at ImCU admission, resulting in a minimal score of 0 and a maximal score of 84. The mean ± standard deviation score in the development group was 22.1 ± 15.2.). The AUROC was 0.843 (95% CI 0.805–0.881), showing good discrimination.

Probability of in-hospital mortality based on the ImCUSS was estimated using logistic regression. The scale of the score was determined to be linear in the logit using fractional polynomials. The coefficient for the score is 0.099 (standard error 0.010, OR 1.10 with 95% CI 1.08–1.13, p<0.001), and the model intercept -4.111 (standard error 0.342, p<0.001). The Hosmer-Lemeshow GOF p value is 0.111. In order to estimate the probability of in-hospital mortality, one would multiply the obtained ImCUSS by the coefficient and then add the intercept. This sum would be exponentiated and then divided by the addition of one plus the exponentiated sum.

$$ImCUSSformula:e^{ImCUSSpoints*0.099–4,111}/\left(1+e^{ImCUSSpoints*0.099–4,111}\right)$$

**Fig 1:**
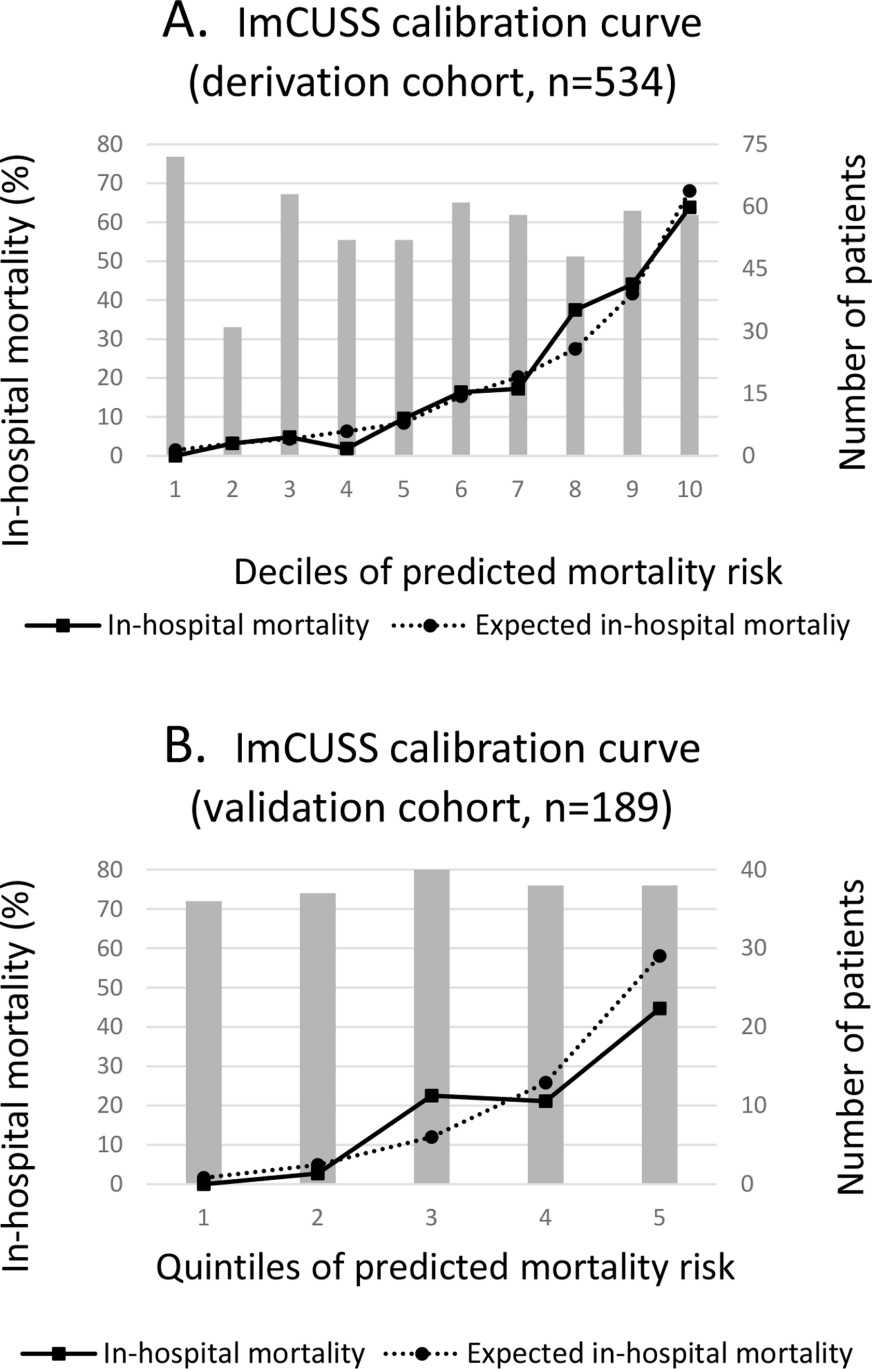
Calibration curves based on Hosmer-Lemeshow goodness-of-fit C test for ImCUSS.

Fig 1A compares observed and predicted mortality (based on the ImCUSS) for all subjects in the development cohort when using deciles of in-hospital mortality risk. These results indicate that estimated and observed hospital mortality pairs were very similar.

**Fig 2:**
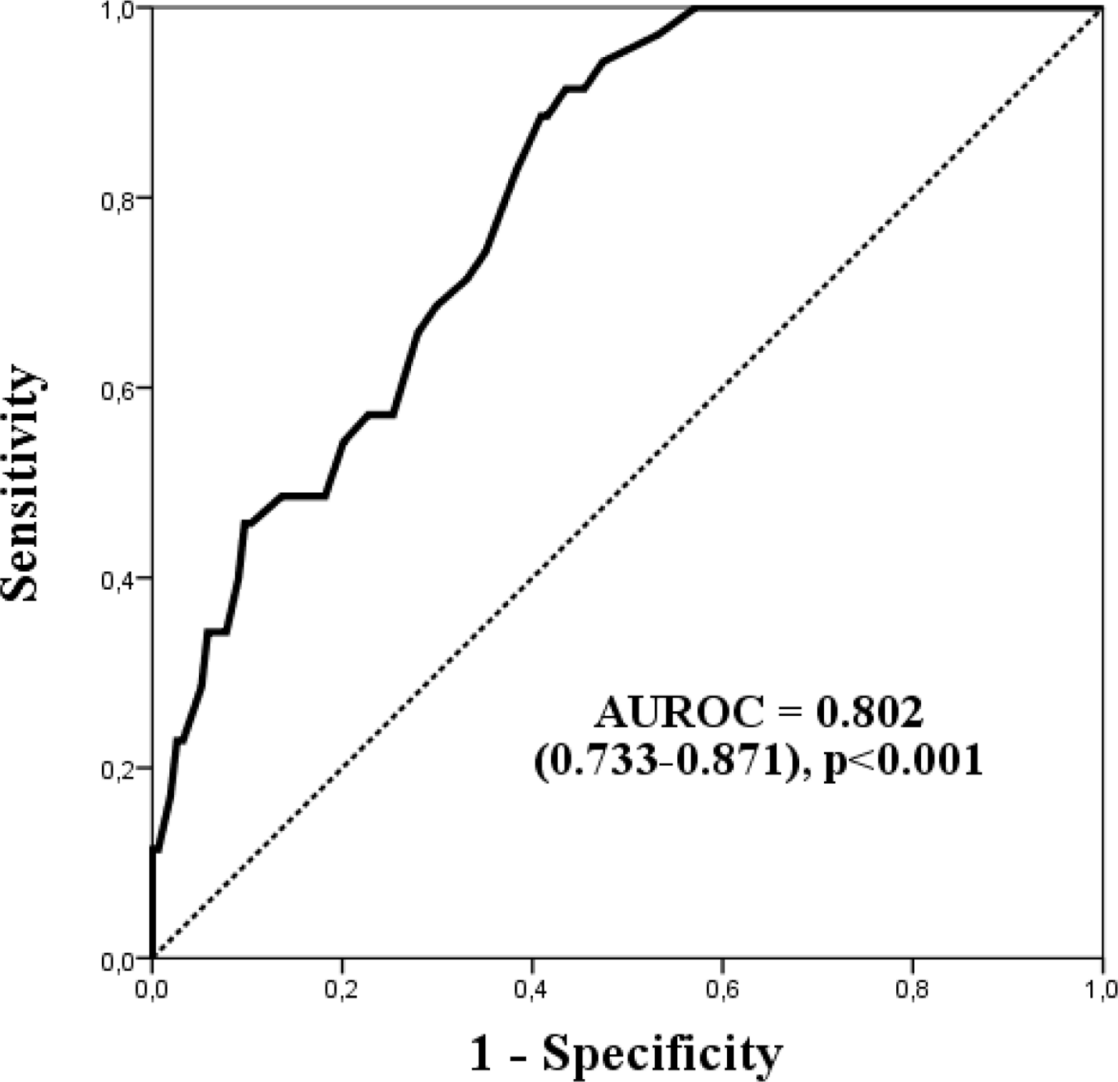
ImCUSS receiver operating characteristic curve (ROC) for mortality in the validation cohort (n = 189).

Performance of the obtained score was studied in the validation cohort (189 patients). The Hosmer-Lemeshow goodness-of-fit showed χ^2^ 8.078 with 7 degrees of freedom and p = 0.326, indicating good calibration. Ability to discriminate in-hospital mortality was also good, with area under receiver operating curve of 0.802 (95% CI 0.733–0.871) (Fig 2). ImCUSS calibration curve for the validation cohort is shown in Fig 1B.

### Comparison of ImCUSS with SAPS II and SAPS 3

The mean ImCUSS (in the validation cohort), SAPS II and SAPS 3 (in the whole cohort) were 21±16.1, 33.1±12.9 and 60.3±14.0 respectively. The expected mortality rates derived from these scores were 20.7±21.9%, 19.2±18.1% and 38.3±23.0%. The observed in-hospital mortality was 18.5% in the validation cohort (35/189) and 19.7% in the whole cohort (146/743), resulting in a standardized mortality ratio (SMR) of 0.89 (95% CI 0.59–1.19) for ImCUSS, 1.03 (95% CI 0.86–1.20) for SAPS II and 0.51 (95% CI 0.43–0.59) for SAPS 3. Interestingly, SAPS 3 clearly overestimated mortality, while SAPS II and ImCUSS showed results closer to the observed mortality.

Performance of the models is presented in Table 3. The scores showed acceptable discrimination, with an AUROC of 0.802 (95% CI 0.733–0.871) for ImCUSS, 0.777 (95% CI 0.737–0.817) for SAPS 2 and 0.793 (95% CI 0.756–0.831) for SAPS 3. The scores also showed similar calibration performance based on the Hosmer-Lemeshow GOF C-test: χ^2^ = 8.708 and p = 0.326 for ImCUSS, χ^2^ = 10.045 and p = 0.262 for SAPS II, and χ^2^ = 4.825 and p = 0.776 for SAPS 3. Integrated discrimination improvement analysis showed p values of 0.898 and 0.231 when comparing performance of ImCUSS versus SAPS II and SAPS 3 scores.

**Table 3:** Performance of ImCUSS, SAPS II and SAPS 3.

	Score	Predicted mortality	SMR * (95% CI)	GOF ₸ χ^2^	GOF ₸ p	AUROC ³ (95% CI)
ImCUSS ^1^	21.9 ± 16.1	20.7 ± 21.9	0.89 (0.59–1.19)	8.078	0.326	0.802 (0.733–0.871)
SAPS II ^2^	33.1 ± 12.9	19.2 ± 18.1	1.03 (0.86–1.20)	10.045	0.262	0.777 (0.737–0.817)
SAPS 3 ^2^	60.3 ± 14.0	38.3 ± 23.0	0.51 (0.43–0.59)	4.825	0.776	0.793 (0.756–0.831)

* SMR: Standardized Mortality Ratio.

^₸^ GOF: Goodness-Of-Fit.

^3^ AUROC: Area Under Receiver Operating Characteristic Curve

^1^ Performance in the validation cohort (n = 189).

^2^ Performance in the whole cohort (n = 743).

### Clinical application of ImCUSS

In an effort to simplify the clinical application, sensitivity analysis was performed to find critical values which could classify patients in statistically different mortality groups. ImCUss was divided in 5 mortality groups; their cut-off values and mean probabilities of in-hospital death are shown in Table 4.

**Table 4:** Clinical application of ImCUSS.

ImCUSS—Points	≤16	17–20	21–30	31–41	≥42
Number of patients	291	69	159	142	82
Observed mortality (%)	2.1 (6/291)	10.1 (7/69)	20.1 (32/159)	36.6 (52/142)	59.8 (49/82)
Mean expected mortality rate (%)	3.71	8.98	16.77	35.37	67.91
ICU transfer in the first 48 hours ^1^ (%)	2.6 (7/272)	3.3 (2/61)	7.4 (9/122)	11.7 (12/103)	38.5 (15/39)

^1^ data regarding ICU (intensive care unit) transfer in the first 48 hours after ImCU (intermediate care unit) admission were calculated considering only patients suitable for transfer to a higher level of care in case of worsening (thus excluding patients with do-not-resuscitate orders at ImCU admission).

Additionally, we decided to study whether ImCUSS could also be useful to identify patients who may benefit of a direct ICU admission in those subjects suitable for transfer to a higher level of care in case of worsening (thus excluding patients with DNR orders at ImCU admission). Transfer to a higher level of care in the first 48 hours after ImCU admission may reflect inappropriate triage, and obviously warrants further consideration. Only 45/597 patients in our series were discharged to the ICU in the first 48 hours after ImCU admission, thus reflecting adequate global triage. Nevertheless, when studying this endpoint in the different mortality groups previously described (Table 4), we observe that patients with an ImCUSS ≥42 should be considered for direct ICU admission (eventually, 38.5% of them would need transfer to this unit in the following 48 hours in case they are admitted to the ImCU).

## Discussion

ImCUs are an attractive alternative for the management of complex patients, in need of special monitoring and nursing care. Concerning the high costs of the traditional ICU and the limited resources of the general wards, multipurpose ImCUs were developed to try to give rational and proportional care. Description of this population is necessary and must be based on accurate severity scoring. Multiple severity scores have been designed and widely described in ICU patients: APACHE II, APACHE III, SAPS II, SAPS 3, SOFA, APACHE IV, MPM II (0 and 24), among others [12–21]. However, the information is scarce and the ability of these scores to accurately and reliably predict mortality in ImCU is inconsistent.

In 1998, Auriant et al [8] described the performance of SAPS II in a cohort of 433 patients, showing good discriminant power (AUROC 0.85) and calibration (C = 2.4, p<0.5), with an SMR of 0.93. Posteriorly, Ip et al [9] described the application of the APACHE II and SAPS scores in a geriatric ImCU cohort of 150 patients. Both scores showed acceptable performance regarding observed to expected mortality ratios (correlation coefficients of 0.97 and 0.92 respectively), but there is no data about calibration. In 2006, Torres et al [11] described the mortality risk of a cohort of 412 patients, using the APACHE II and TISS-28 scores. Mortality discrimination was acceptable (AUROC 0.77 and 0.88 respectively), and the scores showed statistical significant correlation with mortality in the logistic regression analysis. Unfortunately, the scores were not calibrated. Recently, our group described the performance of SAPS II and SAPS 3 in a cohort of 607 patients [5]. Both scores showed acceptable discrimination (AUROC 0.76 and 0.75 respectively) and calibration (χ^2^ = 12.9, p = 0.113 for SAPS II and χ^2^ = 4.07, p = 0.851for SAPS 3). Nonetheless, in this study SAPS 3 clearly overestimated mortality (SMR = 0.56), while the oldest version SAPS II showed better discrimination in terms of SMR, with results closer to the observed mortality (SMR = 0.87). These conflicting results, and the insufficient information, emphasize the need to find more reliable and accurate scores for the ImCU setting. In this context, the present study describes the design and performance of a new severity-of-illness model, which was developed using simple variables, easily obtained at patient admission to the ImCU. To the best of our knowledge, this is the first description of a new score, specifically designed for intermediate care. Moreover, it included the largest population in this setting.

Data regarding patient characteristics and overall mortality are similar to those previously published by our group [1,5]. Observed in-hospital mortality was 19.7%, different to that of other ImCUs and similar to that observed in ICU population, with very high risk for major complications and mortality. The contribution of oncologic patients (284/753), most of them with advanced disease (194/284 had metastatic cancer and/or haematological cancer) and elevated severity-of-illness scoring system predicted risk of death (with mean expected mortality rates of 24.5, 49.5 and 31.1% based on SAPS II, SAPS 3 and ImCUSS scores) probably contributed to the high acuity of our population. Additionally, a total of 146/793 patients had DNR-orders at admission to the ImCU.

The ImCUSS was designed based on 554 patients, and internally validated in 189 additional patients. Its simplicity is remarkable, as it only contains 9 dichotomic variables that are easily obtained in common practice. Four of them are related to previous history (length of in-hospital stay ≥7 days, health-care related infection, metastatic cancer and immunosuppressive therapy), two of them are bed-side obtained (Glasgow Comma Scale ≤12 and need of non-invasive mechanical ventilation), and the remaining three variables depend on routine blood tests (platelets ≤50000/mcL, urea ≥0.6 g/L and bilirubin ≥4 mg/dL) (Table 2). Consequently, its application could reduce the collection burden and potential errors associated to the time-consuming traditional scores. Despite the small number of patients, the ImCUSS showed adequate calibration and discrimination (Figs 1 and 2, and Table 3). Moreover, limiting acquisition of data to patient admission should minimise the impact of mortality overestimation associated with the occurrence of more abnormal physiologic values during the first 24 hours of ImCU stay. This, so-called Boyd and Grounds effect [23], might affect mortality prediction in scores like SAPS II, because the increase in computed severity illness and predicted mortality could be due to suboptimal care, more than to an increase in disease severity.

When we analyse the performance of the traditional scores in our population, it is also noteworthy the mortality overprediction based on SAPS 3 score (SMR = 0.51) compared with the ImCUSS (SMR = 0.89) and SAPS II (SMR = 1.03). In this setting, the differences in the case-mix profiles with the original cohort (SAPS 3), and the simplicity of the two other scores could explain this potential mortality overestimation [24]. Recent external validation studies showed similar results for SAPS 3 [5,25–26]. Nonetheless, although the global performance of the ImCUSS, SAPS II and 3 did not revealed meaningful differences, three aspects must be highlighted. First of all, ImCUSS is specifically designed for multipurpose ImCUs. Secondly, collection of data is made at admission to the ImCU (SAPS II includes variables collected in the first 24 hours after admission). Finally, its simplicity could be a remarkable advantage for ImCU mortality prediction.

Clinical application of ImCUSS is evident, as it provides prognostic information and an estimated in-hospital mortality rate. In addition, it may also be useful to identify patients who may benefit of a direct ICU admission, as we have previously observed in Table 4.

However, in the present study some limitations must be addressed. Lack of data regarding post-ImCU care and its impact in hospital-derived outcomes is shared by all previous scoring systems [27]. The limited number of patients derived from a single centre multipurpose ImCU, and even more restricted samples of various subgroups of the population (patients with AIDS were absent), could interfere with the evaluation of the uniformity-of-fit among different expected mortality subgroups and also limit the applicability of the new score in these specific populations. Furthermore, as previously explained, the case-mix of our cohort (advanced age, high prevalence of cancer, deteriorated functional status and DNR orders) may differ from that in other ImCUs, limiting the extrapolation of the results. Accordingly, we need larger, prospective and well-designed studies with external validation cohorts, before the routine application of the ImCUSS.

## Conclusions

ImCUSS is a new score specially designed for intermediate care, based on simple and easy to calculate variables at admission, and with a notable performance in terms of calibration and mortality discrimination.
